# Do patients bypass primary care for common health problems under a free-access system? Experience of Taiwan

**DOI:** 10.1186/s12913-020-05908-w

**Published:** 2020-11-18

**Authors:** Li-Lin Liang, Nicole Huang, Yi-Jung Shen, Annie Yu-An Chen, Yiing-Jenq Chou

**Affiliations:** 1 Department of Business Management, National Sun Yat-sen University, No. 70, Lienhai Rd, Kaohsiung, 804 Taiwan; 2 Institute of Hospital and Health Care Administration, National Yang-Ming University, No.155, Section 2, Li-Nong Street, Taipei, 112 Taiwan; 3 RAND Corporation, 1766 Main Street, Santa Monica, CA USA; 4 Pardee RAND Graduate School, 1766 Main Street, Santa Monica, CA USA; 5 Institute of Public Health, National Yang-Ming University, No.155, Sec. 2, Li-Nong St., Beitou Dist, Taipei, 112 Taiwan

**Keywords:** Outpatient visits, Referral, Health care delivery, Primary care, Family physicians

## Abstract

**Background:**

A common challenge for free-access systems is that people may bypass primary care and seek secondary care through self-referral. Taiwan’s government has undertaken various initiatives to mitigate bypass; however, little is known about whether the bypass trend has decreased over time. This study examined the extent to which patients bypass primary care for treatment of common diseases and factors associated with bypass under Taiwan’s free-access system.

**Methods:**

This repeated cross-sectional study analyzed data from Taiwan’s National Health Insurance Research Database. A random sample of 1 million enrollees was drawn repeatedly from the insured population during 2000–2017. To capture visits beyond the community level, the bypass rate was defined as the proportion of self-referred visits to the top two levels of providers, namely academic medical centers and regional hospitals, among all visits to all providers. Subgroup analyses were conducted for visits with a single diagnosis. Logistic regressions were used to investigate factors associated with bypass.

**Results:**

The standardized bypass rate for all diseases analyzed exhibited a decreasing trend. In 2017, it was low for common cold (0.7–1.3%), moderate for hypertension (14.0–29.5%), but still high for diabetes (32.0–47.0%). Moreover, the likelihood of bypass was higher for male, patients with higher salaries or comorbidities, and in areas with more physicians practicing in large hospitals or less physicians working in primary care facilities.

**Conclusions:**

Although the bypass trend has decreased over time, continuing efforts may be required to reduce bypass associated with chronic diseases. Both patient sociodemographic and market characteristics were associated with the likelihood of bypass. These results may help policymakers to develop strategies to mitigate bypass.

**Supplementary Information:**

The online version contains supplementary material available at 10.1186/s12913-020-05908-w.

## Introduction

The principles of primary care, namely, first-contact, continuous, coordinated, and comprehensive care, have contributed significantly to population health and health care systems [1, 2]. First-contact care can be achieved by introducing a gatekeeping system in which access to secondary care is only available through referrals from primary care physicians (PCPs) [3]. Another approach to facilitating first-contact care is increasing the cost for visits to specialists without a referral [4]. When people have to bear additional costs to bypass primary care, the financial incentive may prompt them to visit PCPs first. Compared with gatekeeping regulations, allowing choice between levels of care enhances patient satisfaction and empowerment [5, 6]. However, such freedom may cause a violation of the first-contact principle when financial incentives fail to refrain people from bypassing primary care.

Bypassing primary care for common diseases or minor illnesses is particularly worrisome, because it undermines primary care functions, crowds out resources for patients in need of high-level care, and causes physician burnout in hospitals [7–9]. The problems have motivated the current study to investigate bypass for treatment of common health concerns in Taiwan. In a tiered health care delivery system, primary care is for frequent health problems, secondary care for assisting PCPs with diagnostic and therapeutic dilemmas, and tertiary care for uncommon diseases that cannot be competently managed by PCPs [10]. This division of roles is the foundation for efficient and effective health care services [11]. However, it may not be fully realized when people with minor illnesses were treated by secondary or tertiary care providers.

Globally, reforms have been introduced to reduce bypass, including those in France [12], Germany [13], and Belgium [14]. Most initiatives did not lead to imposition of gatekeeping regulations, but resulted in introduction of stronger incentives for patients to use primary care, with negligible effects [15]. Other countries that traditionally provide unrestricted access to specialists, such as China [16] and Japan [17], only piloted the gatekeeping function of PCPs in small areas, without extending it to the entire nation. Considering these challenges, understanding bypass behavior is critical to devise effective policies for establishing a tiered delivery system.

Most studies on access policies pertain to countries with gatekeeping systems [18, 19]; evidence regarding bypass for common diseases is limited. The few studies on bypass behavior have investigated reasons for bypass or self-referral in the Netherlands [20], Japan [21], Israel [22], the United States [23–26], and African countries [27, 28]. The obtained or predicted bypass rates ranged from 13.7% in Japan [21] to 59% in Tanzania [27]. These results imply that bypass behavior is context dependent and that country-specific analyses are necessary to gain deeper insights into its prevalence and nature.

**Fig. 1:**
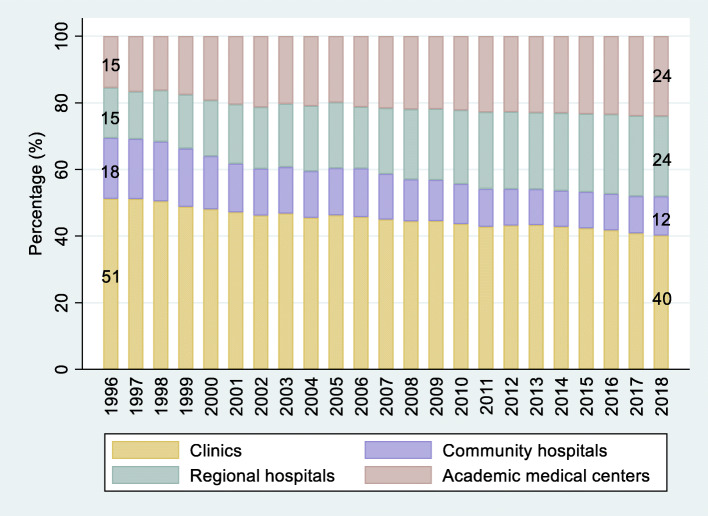
Total outpatient expenditure by provider level, 1996–2018

In this study, we seek to determine the prevalence and trend of bypass for common diseases, as well as factors associated with bypass in Taiwan. Since the introduction of Taiwan’s National Health Insurance (NHI) program in 1995, the NHI Administration (NHIA) has been the single payer in the health care market [29]. Taiwanese people enjoy complete freedom in choosing between health care providers at all levels. Provider choice and high accessibility of care are among the key factors leading to high public satisfaction rates [7]. However, the insurance system has been marred by the problem of allocation of disproportional resources toward large hospitals. Between 1996 and 2018 (Fig. 1), the proportion of outpatient expenditure allocated to the top two levels of providers, namely academic medical centers and regional hospitals (collectively termed large or high-level hospitals), increased from 31 to 48%. During this period, the proportion of outpatient expenditure allocated to the bottom two levels of providers, namely community hospitals and clinics, decreased from 69 to 52%. In terms of the amount of outpatient expenditure, between 1996 and 2018, it increased by approximately 252% in high-level hospitals, from 45.8 to 161.2 billon Taiwanese dollars (in constant 1996 prices) [30].

Research showed that disproportional allocation of expenditures was primarily due to the rapid increase in outpatient visits in large hospitals [31]. There have been concerns about the high percentage of outpatient services delivered by large hospitals in Taiwan [32]. To divert patients to providers at lower levels, the NHIA undertook various initiatives, such as establishing a national electronic referral system, introducing the Family Doctor Plan to promote integrated care [33], increasing copayments for patients visiting hospitals through self-referral, and imposing a strict regulation requiring large hospitals to reduce the number of outpatients seen by 2% annually [29].

To date, little is known about the prevalence and trend of bypass in Taiwan, and in particular, whether NHIA reforms have any effect on mitigating bypass. Various factors may be related to bypass, including patient preference, high accessibility of secondary care, and medical needs associated with the aging population and disease severity [31]. This study investigated related factors, and revealed trends of bypass for common diseases over 2000–2017. Results from this study will shed light on bypass problems and share lessons with countries facing similar problems as Taiwan.

## Methods

### Study design and sample

This study applied a repeated cross-sectional design and used data provided by the National Health Insurance Research Database (NHIRD). NHIRD contains comprehensive data on enrollees’ sociodemographic characteristics, including sex, date of birth, salary and occupation, as well as outpatient and inpatient claims. Claims data include primary and secondary diagnoses, date of visit or hospitalization, medical procedures, prescriptions of drugs, and itemized expenditures. NHIRD used the International Classification of Diseases, 9th Revision, Clinical Modification (ICD-9-CM) to recode diagnoses until January 2016, when the 10th Revision (ICD-10-CM) was introduced.

The study sample was composed of a random sample of 1 million NHI enrollees repeatedly drawn from the insured population every 5 years from 2000 to 2015 as well as in 2017. To explore distributions of outpatient visits, we calculated the numbers of visits made by these representative samples to four levels of providers respectively: academic medical centers, regional hospitals, community hospitals, and clinics. Visits to dental care and traditional Chinese medicine clinics or departments, as well as emergency and accident departments were excluded.

### Descriptive analyses of bypass rates for common diseases

We defined the bypass rate, or the prevalence of bypass, as the proportion of self-referred visits to the top two levels of providers among all visits to all providers. We excluded from the analyses visits to large hospitals through physician referrals, follow-up visits to the same doctor and with the same diagnosis, and visits for refilling prescriptions for chronic illnesses. Clinics and community hospitals are seen as a broad category of primary care facilities in this study, since they are intended for managing common health problems occurring in local populations. For example, under the Family Doctor Plan, NHIA encouraged clinics and community hospitals to form community health care groups for providing comprehensive and continuous care near people’s homes [29].

Patients who did not visit clinics or community hospitals first are regarded as having bypassed primary care in this study. Thus it is necessary to focus on people who were relatively healthy and could be well treated at primary care settings. Accordingly, we excluded patients with catastrophic illnesses (e.g. cancer, end-stage renal disease) identified through Registry for Catastrophic Illness Patient Database in NHIRD, and conducted subgroup analyses for outpatient visits that had a single diagnosis.

Analyses of bypass were conducted for two common chronic diseases, diabetes and hypertension, and one acute condition, common cold. These diseases were selected because of their high patient volume (as a primary diagnosis) in Taiwan, and because they can be adequately managed and treated cost-effectively in primary care settings. We identified disease-specific visits based on their primary diagnoses recorded in the NHI claims data. Additional file 1: Appendix A provides the list of ICD-9/10-CM codes for diabetes, hypertension, and common cold used in this study. Outpatient visits in all years were standardized by holding the age distribution (< 18, 18–64, > = 65 years old) constant, using year 2000 as the base year. See Additional file 1: Appendix B for the standardization method.

### Regression analyses for factors associated with bypass

Factors associated with the likelihood of bypass were investigated by applying logistic regressions. Sample for disease-specific visits discussed previously was used. The outcome variable was a bypass indicator that took on the value of 1 if the visit was at the top two levels of providers, and 0 otherwise. The predictors were the patient’s sex, age, socioeconomic status, and comorbidities, as well as market characteristics and time trends. Socioeconomic status was determined by the enrollee’s salary and occupation based on which the NHI premium was determined. Comorbidities included hyperlipidemia, hypertension, coronary artery disease, cerebrovascular disease, chronic obstructive pulmonary disease (COPD), asthma, chronic liver disease, and chronic kidney disease. Patients were regarded as having a confirmed comorbidity if the corresponding diagnosis code appeared as either a primary or secondary diagnosis at least three times in NHIRD. The related ICD-9/10-CM codes are provided in Additional file 1: Appendix A.

Market characteristics are geographical areas where health care providers were located, and the number of physicians practicing in the top two levels (academic medical center and regional hospitals) and bottom two levels (community hospitals and clinics) of institutions per 10,000 local residents, respectively. Time trends were a set of indicators for year 2005, 2010, 2015, and 2017, respectively. All statistical analyses were performed using SAS software (version 9.4).

## Results

Table 1 presents the characteristics of the sample population. Between 2000 and 2017, the percentage of patients aged younger than 18 years decreased and that of patients aged 65 years or older increased. The percentage of people whose salaries were classified as median (20,000–39,999 NTD) and high (≧40,000 NTD) doubled, and those classified as low (< 20,000) decreased due to increases in minimum wages. Moreover, the percentage of people who had comorbidities increased significantly.

**Table 1:** Characteristics of sample population

Year	2000	2005	2010	2015	2017
*N* = 1,000,000	%	%	%	%	%
Sex					
Male	50.6	50.3	49.8	49.6	49.6
Female	49.4	49.7	50.3	50.4	50.4
Age					
< 18	24.0	21.1	18.0	15.5	14.6
18 ~ 64	66.6	68.4	70.6	70.8	70.4
> =65	9.4	10.6	11.4	13.7	15.0
Socioeconomic status, NTD					
< 20,000	31.8	24.8	13.2	0.5	0.2
20,000-39,999	24.8	25.6	36.4	47.4	47.4
≥ 40,000	12.2	19.0	20.7	24.3	25.6
Farmers and fishers	14.9	13.7	12.1	10.3	9.5
Others	16.4	16.9	17.6	17.6	17.4
Comorbidities					
Diabetes	1.1	1.8	2.4	4.0	4.4
Hyperlipidemia	1.1	2.4	4.3	8.0	8.7
Hypertension	2.5	3.6	4.7	7.0	7.6
Coronary artery disease	1.0	1.3	1.6	2.2	2.2
Cerebrovascular disease	0.6	0.7	0.8	1.3	1.1
COPD	0.6	0.6	0.5	0.8	0.8
Asthma	0.5	0.5	0.5	0.7	0.7
Chronic liver disease	0.7	0.9	1.0	1.6	2.0
Chronic kidney disease	0.3	0.3	0.4	1.0	1.5

**Fig. 2:**
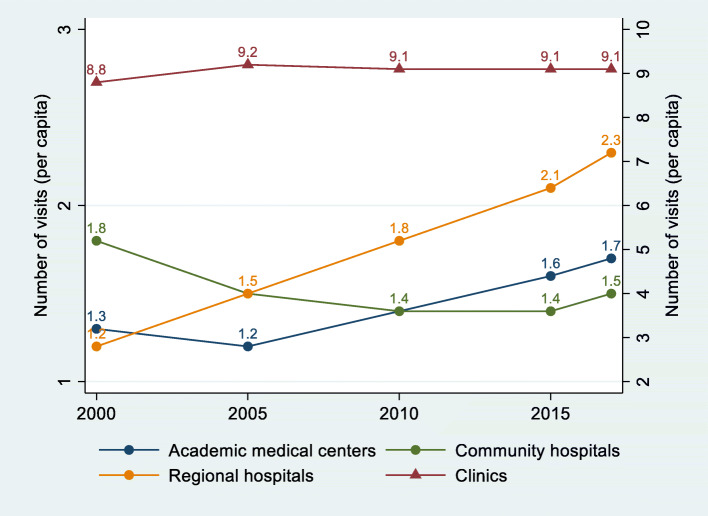
Number of visits per capita by provider level, 2000–2017

Figure 2 presents the per capita visits made by the sample population across four provider levels. The left y-axis denotes the number of visits for academic, regional and community hospitals; the right y-axis denotes the number of visits for clinics. Between 2000 and 2017, the per capita visits decreased for community hospitals (from 1.8 to 1.5), with a growth rate of − 19.6%, and remained relatively stable for clinics (from 8.8 to 9.1), with a growth rate of 2.8%. During the same period, regional hospitals had an exceptionally high growth rate of per capita visits (nearly doubled, from 1.2 to 2.3), followed by academic medical centers (from 1.3 to 1.7, or 34.4%). This indicates a disproportional increase in outpatient volume in large hospitals.

Table 2 displays distributions of disease-specific visits and the corresponding bypass rates. Results for visits with a single diagnosis are also reported. For all visits, in 2017, the bypass rate was 47.0% for diabetes, 29.5% for hypertension, and 1.3% for common cold. For single-diagnosis visits, the bypass rate was lower: 32.0% for diabetes, 14.0% for hypertension, and 0.7% for common cold. Moreover, comparing the data from 2017 with those from 2000 revealed that the bypass rate for visits with a single diagnosis of diabetes and hypertension decreased by 0.8 and 9.4 percentage points, respectively. However, during the same period, all visits for diabetes increased by 2.9 percentage points, mostly due to an increase in visits to regional hospitals.

**Table 2:** Outpatient visits for common diseases across provider levels, 2000–2017

	All visits						Visits with single diagnosis					
	2000	2005	2010	2015	2017	Difference (2017 vs. 2000)	2000	2005	2010	2015	2017	Difference (2017 vs. 2000)
	%	%	%	%	%		%	%	%	%	%	
**Diabetes**												
Academic medical centers	23.2	19.3	20.2	19.4	19.0	−4.2	16.7	12.2	12.3	11.3	11.9	−4.8
Regional hospitals	20.9	24.3	26.6	27.6	28.0	7.1	16.1	16.1	15.7	19.1	20.1	4.0
Community hospitals	23.2	19.4	15.8	14.9	14.7	−8.5	20.1	14.0	9.2	8.8	9.1	−11.0
Clinics	32.7	36.9	37.4	38.2	38.3	5.6	47.0	57.6	62.9	60.9	58.9	11.9
Bypass rate^a^	44.1	43.6	46.8	47.0	47.0	2.9	32.8	28.4	28.0	30.3	32.0	−0.8
**Hypertension**												
Academic medical centers	16.2	13.1	13.5	13.4	10.9	−5.3	12.5	7.6	5.4	5.0	5.0	−7.5
Regional hospitals	17.7	17.0	18.0	18.9	18.7	1.0	10.9	8.2	7.6	8.0	9.0	−1.9
Community hospitals	23.1	17.1	12.7	12.1	12.4	−10.7	15.5	9.5	6.5	6.0	6.4	−9.1
Clinics	43.0	52.7	55.9	55.6	58.1	15.1	61.1	74.7	80.5	81.0	79.6	18.5
Bypass rate	33.9	30.2	31.4	32.3	29.5	−4.4	23.4	15.8	13.0	13.1	14.0	−9.4
**Common cold**												
Academic medical centers	0.5	0.4	0.3	0.3	0.3	−0.2	0.2	0.2	0.2	0.2	0.1	−0.1
Regional hospitals	1.0	0.8	0.9	0.9	1.0	0.0	0.5	0.4	0.5	0.5	0.5	0.0
Community hospitals	4.5	2.9	2.3	1.9	1.8	−2.7	2.3	1.4	1.1	1.1	1.1	−1.2
Clinics	94.1	95.9	96.5	96.9	97.0	2.9	97.0	98.0	98.2	98.3	98.3	1.3
Bypass rate	1.4	1.1	1.2	1.2	1.3	−0.1	0.7	0.6	0.6	0.6	0.7	0.0

^a^Bypass rate was calculated as the proportion of self-referred visits to academic medical centers and regional hospitals among all visits to all providers. The sample size (number of visits) varied across diseases and over time. For visits without secondary diagnoses, the sample size for diabetes, hypertension and common cold was 4527 – 6068, 12,578 – 21,327, and 149,468 – 341,825, respectively. For visits with secondary diagnoses, the sample size for diabetes, hypertension and common cold was 20,404 – 45,294, 41,919 – 73,816, and 205,269 – 388,925, respectively

Tables 3 and 4 summarizes the results of logistic regressions for all visits and visits with a single diagnosis, respectively. All models indicate that females were less likely to bypass primary care than males (OR 0.81–0.95, *P* < 0.001), and patients with high salaries (≧40,000 NT$) had a higher likelihood of bypass than those with low salaries (< 20,000 NT$) (OR 1.12–1.67, *P* < 0.001). Furthermore, comorbidities were positively associated with the likelihood of bypass. For example, Table 3 shows that the likelihood of bypass was higher for people with coronary artery disease (OR 1.12–1.61, *P* < 0.001), cerebrovascular disease (OR 1.67–2.43, *P* < 0.001), COPD (OR 1.26–3.64, *P* < 0.001), chronic liver disease (OR 1.19–1.55, *P* < 0.001), and chronic kidney disease (OR 1.45–1.88, *P* < 0.001). Interestingly, after controlling for sex, socioeconomic status, comorbidities and other variables, age appeared to be a less important predictor, with odd ratios near one.

**Table 3:** Logistic regressions for the likelihood of bypass: all visits

	Diabetes				Hypertension				Common cold			
Predictors	OR^a^	SE^b^	P	95% CI	OR	SE	P	95% CI	OR	SE	P	95% CI
Age	1.00	0.00	0.037	1.00–1.00	1.00	0.00	< 0.001	1.00–1.00	0.98	0.00	< 0.001	0.98–0.98
Female	0.93	0.01	< 0.001	0.91–0.95	0.95	0.01	< 0.001	0.93–0.97	0.92	0.01	< 0.001	0.89–0.95
Socioeconomic status, NTD												
< 20,000	1.00	ref^c^			1.00	ref			1.00	ref		
20,000-39,999	0.98	0.02	0.403	0.94–1.03	0.95	0.02	0.009	0.92–0.99	1.22	0.03	< 0.001	1.16–1.28
≥ 40,000	1.14	0.03	< 0.001	1.09–1.20	1.12	0.02	< 0.001	1.08–1.17	1.43	0.04	< 0.001	1.36–1.51
Farmers and fishers	0.84	0.02	< 0.001	0.80–0.88	0.78	0.02	< 0.001	0.75–0.81	1.25	0.04	< 0.001	1.18–1.33
Others	1.06	0.03	0.015	1.01–1.11	1.09	0.02	< 0.001	1.05–1.13	1.96	0.05	< 0.001	1.86–2.07
Comorbidities												
Diabetes	n/a	n/a	n/a	n/a	1.31	0.02	< 0.001	1.28–1.34	1.21	0.06	< 0.001	1.10–1.33
Hyperlipidemia	1.30	0.01	< 0.001	1.27–1.33	1.88	0.02	< 0.001	1.84–1.91	1.11	0.04	0.012	1.02–1.20
Hypertension	1.13	0.01	< 0.001	1.11–1.16	n/a	n/a	n/a	n/a	2.10	0.08	< 0.001	1.96–2.25
Coronary artery disease	1.12	0.02	< 0.001	1.08–1.15	1.61	0.02	< 0.001	1.57–1.65	1.49	0.08	< 0.001	1.35–1.65
Cerebrovascular disease	1.67	0.04	< 0.001	1.59–1.74	1.96	0.04	< 0.001	1.89–2.03	2.43	0.15	< 0.001	2.15–2.75
COPD^d^	1.26	0.04	< 0.001	1.18–1.34	1.54	0.04	< 0.001	1.47–1.61	3.64	0.18	< 0.001	3.30–4.01
Asthma	0.80	0.04	< 0.001	0.73–0.87	1.08	0.04	0.025	1.01–1.15	5.35	0.17	< 0.001	5.03–5.70
Chronic liver disease	1.19	0.02	< 0.001	1.15–1.23	1.19	0.02	< 0.001	1.15–1.24	1.55	0.09	< 0.001	1.38–1.75
Chronic kidney disease	1.45	0.03	< 0.001	1.38–1.51	1.74	0.04	< 0.001	1.66–1.82	1.88	0.14	< 0.001	1.62–2.18
Geographical area												
Taipei	1.00	ref			1.00	ref			1.00	ref		
North	0.93	0.02	< 0.001	0.90–0.96	0.91	0.01	< 0.001	0.89–0.94	0.90	0.03	< 0.001	0.85–0.95
Central	0.89	0.01	< 0.001	0.86–0.92	0.78	0.01	< 0.001	0.76–0.80	1.18	0.03	< 0.001	1.13–1.24
South	1.01	0.02	0.455	0.98–1.05	0.96	0.01	0.005	0.93–0.99	1.13	0.03	< 0.001	1.07–1.19
Kao-Ping	0.87	0.02	< 0.001	0.84–0.90	0.83	0.01	< 0.001	0.81–0.85	0.88	0.03	< 0.001	0.83–0.93
East	0.51	0.02	< 0.001	0.47–0.54	0.56	0.02	< 0.001	0.53–0.59	1.66	0.07	< 0.001	1.53–1.81
Number of physicians practicing in academic medical centers and regional hospitals (per 10,000 residents)	1.12	0.00	< 0.001	1.11–1.12	1.11	0.00	< 0.001	1.11–1.12	1.10	0.00	< 0.001	1.09–1.10
Number of physicians practicing in community hospitals and clinics (per 10,000 residents)	0.92	0.00	< 0.001	0.92–0.93	0.95	0.00	< 0.001	0.94–0.95	1.02	0.01	0.015	1.00–1.03
Year												
2000	1.00	ref			1.00	ref			1.00	ref		
2005	0.98	0.02	0.423	0.95–1.02	0.88	0.01	< 0.001	0.85–0.91	0.73	0.02	< 0.001	0.70–0.77
2010	0.89	0.02	< 0.001	0.86–0.93	0.70	0.01	< 0.001	0.68–0.72	0.67	0.02	< 0.001	0.63–0.70
2015	0.76	0.02	< 0.001	0.73–0.79	0.61	0.01	< 0.001	0.59–0.63	0.56	0.02	< 0.001	0.53–0.59
2017	0.72	0.02	< 0.001	0.69–0.75	0.50	0.01	< 0.001	0.48–0.51	0.52	0.02	< 0.001	0.49–0.56

Note: The sample size (number of visits) for diabetes, hypertension, and common cold was 172,944, 308,743, and 1,389,726, respectively. The dependent variable was dichotomous (1 = visited medical centers or regional hospitals; 0 = visited community hospitals or clinics). ^a^ OR: Odds Ratio. ^b^ SE: Standard Errors. ^c^ ref.: reference category. ^d^ COPD: Chronic Obstruction Pulmonary Disease

**Table 4:** Logistic regressions for the likelihood of bypass: visits with single diagnosis

	Diabetes				Hypertension				Common cold			
Predictors	OR^a^	SE^b^	P	95% CI	OR	SE	P	95% CI	OR	SE	P	95% CI
Age	0.98	0.00	< 0.001	0.98–0.99	0.98	0.00	< 0.001	0.98–0.98	0.98	0.00	< 0.001	0.97–0.98
Female	0.81	0.02	< 0.001	0.76–0.86	0.92	0.02	< 0.001	0.88–0.96	0.95	0.02	0.032	0.91–1.00
Socioeconomic status, NTD												
< 20,000	1.00	ref^c^			1.00	ref			1.00	ref		
20,000-39,999	1.03	0.06	0.623	0.92–1.14	0.99	0.04	0.815	0.92–1.07	1.35	0.05	< 0.001	1.25–1.46
≥ 40,000	1.29	0.07	< 0.001	1.15–1.45	1.21	0.05	< 0.001	1.12–1.31	1.67	0.07	< 0.001	1.54–1.82
Farmers and fishers	0.83	0.05	0.001	0.74–0.93	0.72	0.03	< 0.001	0.66–0.79	1.32	0.07	< 0.001	1.19–1.45
Others	1.27	0.07	< 0.001	1.14–1.42	1.29	0.05	< 0.001	1.19–1.40	2.13	0.09	< 0.001	1.96–2.32
Comorbidities												
Diabetes	n/a	n/a	n/a	n/a	1.19	0.06	0.001	1.08–1.31	1.20	0.11	0.035	1.01–1.43
Hyperlipidemia	1.09	0.04	0.027	1.01–1.17	1.54	0.05	< 0.001	1.44–1.64	1.05	0.08	0.542	0.91–1.21
Hypertension	1.16	0.05	< 0.001	1.07–1.26	n/a	n/a	n/a	n/a	1.90	0.13	< 0.001	1.67–2.17
Coronary artery disease	1.23	0.09	0.003	1.07–1.42	1.23	0.07	< 0.001	1.10–1.38	1.66	0.15	< 0.001	1.39–2.00
Cerebrovascular disease	1.44	0.14	< 0.001	1.20–1.73	1.41	0.11	< 0.001	1.21–1.65	2.54	0.29	< 0.001	2.03–3.17
COPD^d^	1.28	0.17	0.055	0.99–1.65	1.27	0.12	0.012	1.05–1.54	3.08	0.31	< 0.001	2.53–3.75
Asthma	1.05	0.18	0.789	0.75–1.45	1.14	0.13	0.249	0.91–1.44	3.16	0.22	< 0.001	2.76–3.61
Chronic liver disease	1.20	0.08	0.009	1.05–1.38	1.18	0.07	0.01	1.04–1.33	1.56	0.17	< 0.001	1.26–1.93
Chronic kidney disease	1.37	0.14	0.002	1.12–1.67	1.30	0.12	0.006	1.08–1.57	1.36	0.22	0.052	1.00–1.86
Geographical area												
Taipei	1.00	ref			1.00	ref			1.00	ref		
North	0.82	0.04	< 0.001	0.75–0.91	1.03	0.03	0.394	0.96–1.10	0.93	0.04	0.093	0.86–1.01
Central	0.80	0.04	< 0.001	0.73–0.88	0.91	0.03	0.004	0.85–0.97	0.90	0.04	0.006	0.83–0.97
South	0.75	0.04	< 0.001	0.68–0.83	0.91	0.03	0.004	0.85–0.97	1.15	0.05	0.001	1.06–1.26
Kao-Ping	0.71	0.03	< 0.001	0.64–0.78	0.72	0.03	< 0.001	0.67–0.77	0.82	0.04	< 0.001	0.75–0.89
East	0.38	0.04	< 0.001	0.31–0.46	0.53	0.04	< 0.001	0.46–0.61	2.28	0.14	< 0.001	2.03–2.57
Number of physicians practicing in academic medical centers and regional hospitals (per 10,000 residents)	1.12	0.00	< 0.001	1.11–1.13	1.12	0.00	< 0.001	1.12–1.13	1.10	0.00	< 0.001	1.10–1.11
Number of physicians practicing in community hospitals and clinics (per 10,000 residents)	1.01	0.01	0.256	0.99–1.04	0.98	0.01	0.061	0.97–1.00	1.00	0.01	0.651	0.98–1.02
Year												
2000	1.00	ref			1.00	ref			1.00	ref		
2005	0.78	0.04	< 0.001	0.71–0.85	0.63	0.02	< 0.001	0.59–0.67	0.76	0.03	< 0.001	0.71–0.81
2010	0.65	0.03	< 0.001	0.59–0.71	0.44	0.01	< 0.001	0.41–0.47	0.65	0.02	< 0.001	0.61–0.70
2015	0.58	0.03	< 0.001	0.52–0.64	0.34	0.01	< 0.001	0.32–0.37	0.57	0.03	< 0.001	0.52–0.62
2017	0.57	0.03	< 0.001	0.51–0.64	0.35	0.01	< 0.001	0.32–0.37	0.55	0.03	< 0.001	0.50–0.60

Note: The sample size (number of visits) for diabetes, hypertension, and common cold was 26,253, 92,321, and 1,124,721, respectively. The dependent variable was dichotomous (1 = visited medical centers or regional hospitals; 0 = visited community hospitals or clinics). ^a^ OR: Odds Ratio. ^b^ SE: Standard Errors. ^c^ ref.: reference category. ^d^ COPD: Chronic Obstruction Pulmonary Disease

Market characteristics were closely related to the likelihood of bypass. Outpatient visits for diabetes and hypertension in Taipei (reference category) were more likely to bypass primary care; whereas visits for common cold in Eastern areas (OR 1.66–2.28, *P* < 0.001) had a higher likelihood of bypass. A greater number of physicians practicing in large hospitals increased the likelihood of bypass (OR 1.10–1.12, *P* < 0.001); while a greater number of physicians practicing in primary care facilities generally decreased the likelihood of bypass (OR 0.92–1.02, *P* < 0.001).

Notably, indicators for years revealed that the likelihood of bypass decreased over time. For all visits, the odd of bypass for year 2005, 2010, 2015 and 2017 was 0.73–0.88, 0.67–0.89, 0.56–0.76, and 0.50–0.72 times that for year 2000 (*P* < 0.001), respectively. For single-diagnosis visits, the odd of bypass for year 2005, 2010, 2015 and 2017 was 0.63–0.78, 0.44–0.65, 0.34–0.58, and 0.35–0.57 times that for year 2000 (*P* < 0.001), respectively.

## Discussion

This study demonstrated that the prevalence of bypass varied substantially across diseases. In 2017, the bypass rates were considerably low for common cold (0.7–1.3%), moderate for hypertension (14.0–29.5%), and high for diabetes (32.0–47.0%). The wide variation in disease-specific bypass rates indicates that the pervasive view of patients bypassing primary care for minor illnesses is partial, because ailments such as common cold were often treated at clinics. These results also suggest that future policies to mitigate bypass may be refined to target specific diseases with high bypass rates. Around the globe, Korea is similar to Taiwan in its free-access system; research showed that 15% of primary care patients in Korea were treated at hospitals [34]. Both Taiwan and Korea had relatively high bypass rates compared to the United States, where only 3.7–7.2% of enrollees of point-of-service plan self-referred to specialists [35].

Bypass for treatment of chronic conditions can be expected when the perceived benefits are greater than the costs of bypass. In 2017, copayment without a referral was fixed at NT$420 (US$14) per visit for academic medical centers and NT$240 (US$8) for regional hospitals (the 2017 Big Mac price in Taiwan was NT$69, or US$2.3). People are exempted from copayment if they hold a refillable prescription for chronic diseases or certification of vulnerability, e.g., having a disability, being poor, and being a veteran [29]. They are generally high-risk groups of chronic illnesses. Thus the financial burden for visits to large hospitals is considered low for chronic patients in Taiwan.

The perceived benefits of bypassing primary care are largely associated with the greater ability of hospital-employed physicians to mobilize resources. Studies from other countries have consistently demonstrated that more facilities and services provided by specialists are among the main reasons for self-referrals [20, 27]. Compared with clinics and community hospitals, large hospitals in Taiwan have more diagnostic equipment, greater drug variety, and more comprehensive services. Moreover, since 2010, the NHIA has provided financial rewards to hospitals for integrating outpatient sessions across specialty departments [29]. On the other hand, Taiwan’s clinic physicians are not allowed to use hospital facilities to diagnose or treat their patients, which may appear less attractive to patients with chronic conditions who demand comprehensive and coordinated care. Consequently, low-level providers continue to face constraints of physical and human capital in competing with large hospitals in the outpatient care market.

When looking at provider levels, a positive finding is that the share of visits for all diseases analyzed exhibited a decreasing trend for academic medical centers (Table 2). A puzzling finding is that between 2000 and 2017, the share of diabetes visits to regional hospitals did not decrease but instead increased. We conjecture that this may be associated with the 2001 NHI pay-for-performance program introduced for diabetes. The program encourages providers at all levels to enroll patients with diabetes and rewards the providers for conducting regular follow-ups [29]. Regional hospitals enrolled a great proportion of diabetes P4P patients [36], which could have encouraged the bypass behavior in these patients. Streamlining different policies may be required to avoid contradictions of policy goals and to mitigate bypass behavior.

Results from logistic regressions indicated that male patients and patients with higher salaries or comorbidities were more likely to bypass primary care. These findings are consistent with existing evidence that income [25] and severity of illness are positively associated with the bypass [24, 26]. In addition, more physicians working in high-level hospitals increased the odd of bypass; while more physicians working in primary care facilities decreased the likelihood of bypass. These results suggest that physician supply played an important role in determining the observed bypass rates, through changes in access to different levels of care [24], and perhaps also through changes in competition, which could affect patient-perceived quality of care [37].

Crucially, year indicators in logistic regressions revealed that the odd of bypass decreased over time. This result is consistent with descriptive analyses of bypass trends summarized in Table 2. It is interesting to note between 2000 and 2017, enrollees’ salaries and prevalence of comorbidities had increased (Table 1), and the number of physicians in large hospitals grew more (by 75%) than that in primary care facilities (by 43%) [38]. All these factors could increase the likelihood of bypass. However, the bypass rates for common diseases were decreasing. Thus other factors might have contributed to the decreasing trends, such as the accumulated effects of polices for diverting patients to primary care.

This study has several limitations. First, we only selected three common diseases for investigating the bypass rate. Future studies may consider looking at other diseases. Second, when a doctor’s referral was not recorded in the NHI referral system, such visit would be classified as self-referral in this study. We expected the problem to be lessened over time because NHIA has continued to promote the use of referral system. Third, considering that patients may self-refer to hospitals based on PCPs’ verbal advice, it remained unclear the extent to which bypassing primary care was due to patient preference or physicians’ recommendations. Finally, due to data limitations, we were unable to further decompose time-dependent factors associated with decreasing trends of bypass.

## Conclusions

This study demonstrated that the trends of bypassing primary care for treatment of common diseases decreased from 2000. In 2017, the bypass rate was low for common cold, moderate for hypertension, but still high for diabetes. The wide variation in disease-specific bypass rates indicates that policies to mitigate bypass may be refined to target common diseases with high bypass rates, and continuing efforts may be required to reduce bypass associated with chronic diseases. Moreover, the likelihood of bypass was higher for male, patients with higher salaries or comorbidities, and in areas with more physicians practicing in large hospitals or less physicians working in primary care facilities. These results may help developing strategies to mitigate bypass of primary care for common diseases.

## Supplementary Information

Additional file 1: **Appendix A.** Diagnosis codes for diabetes, hypertension, common cold, and comorbidities. **Appendix B.** Standardizing outpatient visits for multiple years.

## Data Availability

This study used data provided by Applied Health Research Data Integration Service, National Health Insurance Administration. The datasets analysed during the current study are not publicly available due to regulations set out by Ministry of Health and Welfare of Taiwan.

## Abbreviations

PCP: Primary care physicians
NHI: National Health Insurance
NHIA: National Health Insurance Administration
