# Supplementary material for: Do patients bypass primary care for common health problems under a free-access system? Experience of Taiwan
Source: BMC Health Serv Res. 2020 Nov 18;20:1050. doi: 10.1186/s12913-020-05908-w (PMC7677770; doi:10.1186/s12913-020-05908-w)
Supplement: Supplementary file 1 — Appendix A. Diagnosis codes for diabetes, hypertension, common cold, and comorbidities. Appendix B. Standardizing outpatient visits for multiple years. [file 12913_2020_5908_MOESM1_ESM.docx]

**Appendix A. Diagnosis codes for diabetes, hypertension, common cold, and comorbidities**

Table A Codes of International Classification of Diseases, 9/10th Revision, Clinical Modification (ICD-9/10-CM)

| **Diseases** | **ICD 9/10** | **Code** |
| --- | --- | --- |
| Diabetes | ICD 9 | First three digits: 250 |
|  | ICD 10 | First three digits: E10-E13 |
| Hypertension | ICD 9 | First three digits: 401-405 |
|  | ICD 10 | First three digits: I10-I15 |
| Common Cold | ICD 9 | First three digits: 460, 462, 464, 465, 466, 487, 490 |
|  | ICD 10 | J00, J060, J069, J069, J09X1, J1000, J1008, J1100, J1108, J09X2, J1001, J101, J111, J09X3, J09X9, J102, J1081, J1082, J1083, J1089, J112, J1181, J1182, J1183, J1189, J028, J029, J040, J0430, J0431, J050, J0410, J0411, J042, J050, J0510, J0511, J050, J208, J209, J210, J211, J218, J219, J40 |
| Hyperlipidemia | ICD 9 | First three digits: 272 |
|  | ICD 10 | First three digits: E78 |
| Coronary artery disease | ICD 9 | First three digits: 410-414 |
|  | ICD 10 | First three digits: I20-I25 |
| Cerebrovascular disease | ICD 9 | First three digits: 430-438 |
|  | ICD 10 | First three digits: I60-I69 |
| Chronic obstructive pulmonary disease | ICD 9 | First three digits: 491, 492, 496 |
|  | ICD 10 | First three digits: J41-J44 |
| Asthma | ICD 9 | First three digits: 493 |
|  | ICD 10 | First three digits: J45 |
| Chronic liver disease | ICD 9 | First three digits: 571 |
|  | ICD 10 | First three digits: K70-K77 |
| Chronic kidney disease | ICD 9 | First three digits: 580-587 |
|  | ICD 10 | First three digits: M05-M08, M30-M36 |

**Appendix B. Standardizing outpatient visits for multiple years**

We adopted SAS STDRATE procedure to standardize the distribution of outpatient visits across provider level. The formula is as follows:

$$r_{dhi}=\frac{\sum_{i} r_{ghi}{\times n}_{g,2000}}{N_{2000}}$$

where $r_{dhi}$ is the standardized rate (percentage of visits) for provider level *h* (1, …, 4) in year *i*. $r_{ghi}$ is the rate for *g^th^* age group in provider level *h* in year *i*. $n_{g,2000}$ is the number of visits in *g^th^* age group in year 2000, and $N_{2000}$ is the total number of visits in year 2000. The standardized percentage of visits used the share of visits made by individual age groups in 2000 as the weights.
